# Methodological Considerations in the Recruitment and Analysis of Schizotypy Samples

**DOI:** 10.3389/fpsyt.2014.00156

**Published:** 2014-11-06

**Authors:** Erica Neill

**Affiliations:** ^1^Monash Alfred Psychiatry Research Centre, Central Clinical School, Monash University, Melbourne, VIC, Australia; ^2^Brain and Psychological Sciences Research Centre, Swinburne University of Technology, Melbourne, VIC, Australia

**Keywords:** schizotypy, methodological considerations, relative status, religion, psychosis over the lifespan

The schizotypy analog allows researchers to control for many of the confounding factors associated with schizophrenia (e.g., medication/illicit drug use and health complications) (1–3). There are, however, still extraneous factors that should be considered when schizotypy samples are employed. The purpose of this article is to highlight some of the areas of consideration including age, education, relative status, abuse history, and religion.

## Age and Education

Most schizotypy studies recruit undergraduate students between the ages of 18–24 years (4–10). The ease with which researchers can access students makes this an attractive option, especially when large samples can be drawn upon from which “high schizotypy” individuals can be sourced. There are, however, well-documented problems with this approach. The age old argument of whether university student performance can be generalized to the population must be considered (11), particularly when the samples are drawn specifically from first-year psychology students. Despite these concerns, there is some evidence that older samples (mean age 40 years) do not necessarily score differently from younger university samples (12). Further research into the effect of education is required; the effects of age are examined next.

## Schizotypy Levels Over the Lifespan

There is evidence that levels of schizotypy change as people age (13–16). Mason (16) reported that while positive schizotypy features decrease with age, introvertive anhedonia (negative symptom analog) actually increases. This may be analogous to the reported reduction in positive symptoms and increased negative symptoms seen in later stages of schizophrenia (17, 18). Thus, recruiting younger groups may result in higher schizotypy scores than would be found in older groups. These younger groups, however, may serve as an appropriate analog only for early stages of schizophrenia. It may be more appropriate to explore issues relating to chronic schizophrenia using an older schizotypy sample. Recruitment of high schizotypy from non-student populations will require a more targeted approach; one method would be to focus on creative individuals including artists and musicians (12) as schizotypy levels are often higher among those in more creative or artistic positions.

## Relative Status

Evidence suggests that schizotypy is higher among relatives of those with psychosis than it is in the general population (19). Studies including relatives have reported that within their high schizotypy groups, schizotypy levels are higher and more variable among those who are relatives (20, 21). Given the difference in performance among relatives, future studies should consider examining relatives separately or at least reporting the breakdown of relatives versus non-relatives among their samples.

## History of Trauma

There is a relationship between physical or sexual abuse and the development of psychosis with some authors going so far as to describe the relationship as “causal” (22). This same relationship has been found in the schizotypy literature (23). Further, evidence suggests that such trauma is associated with the development of specific psychosis symptoms, namely hallucinations (24, 25). Given that the development of specific symptoms has been related to trauma, the same suggestions put forward for relative status (separate analysis for those with a trauma history/report breakdown of trauma vs. non-trauma) should be considered for trauma.

## Religion

Religion is a complex area of investigation in psychosis research. It can be difficult to tease apart delusions with religious content from “healthy” religious belief. This distinction can be even more complicated when examining the schizotypy continuum. In the search for pathology indicators, researchers have noted higher levels of schizotypy among those associated with new religious movements (Hare Krishnas and Druids) compared to levels found among those following mainstream religions (Christianity) (26). Other research has found that religious preoccupation relates to high schizotypy (27). It may be that an association with less mainstream religion and preoccupation may serve as signifiers of more pathological religious belief, which may link to schizotypy. Future research should investigate the relationship between religion and current schizotypy scales to determine whether healthy and more pathological beliefs of a religious nature are contributing to schizotypy scores. Further, those studies that have investigated schizotypy and religion find a gender effect, which should also be considered (27, 28). With one study finding the link between religion and schizotypy only existed for the males in their sample (27). Another paper found that religion related to different aspects of schizotypy depending on gender with men demonstrating a relationship between intrinsic orientation toward religion and more borderline features of schizotypy while for women, there was a relationship between social orientation in religion and paranoia and borderline features (28).

## Dichotomizing Continuous Measures of Schizotypy

In the schizotypy literature, many authors opt to do median splits of their participants to create a “low” and a “high” schizotypy group. This topic will be discussed in more detail in this issue by Mason. As such, this piece will summarize only the main concerns associated with this approach. Firstly, dichotomizing the data results in a loss of power with estimates suggesting that dichotomizing equates to a loss of a third of the data (29). Further, using this method can increase both type I (based on the reduced power) (30) and type II errors (especially when “optimal” or “minimum p value” approaches are taken) (31). Further, it appears that dichotomizing data is more problematic than splitting the sample into more than two groups. Non-linear, especially *U*-shaped relationships, are generally lost using a median split but might be seen if more than two groups are formed (29, 32). Some studies choose to use the upper and lower thirds or top and bottom 25% of their samples to split their groups (33, 34) including one of my own studies: (35). This creates an additional problem, namely, the “low” group. It does not seem likely that the low group is actually representative of the general population. In fact, researchers have suggested that very low scores on the O-LIFE might reflect a tendency to more autistic traits (36). Researchers should consider these significant problems when determining whether to split their data.

## Conclusion

Schizotypy research has been conducted for many years; however, this field is still in its infancy compared to the schizophrenia research field. This area of study is increasing and as such, it is our responsibility to ensure that our research considers the influence of some of the underlying contributions to scores (genetics and trauma) as well as some of the complications, which are often overlooked in the schizophrenia literature (role of religion) as well as ensuring that our studies are well designed and statistically valid.

## Conflict of Interest Statement

The author declares that the research was conducted in the absence of any commercial or financial relationships that could be construed as a potential conflict of interest.
